# Exploring Different Types of Inhibition During Bilingual Language Production

**DOI:** 10.3389/fpsyg.2018.02256

**Published:** 2018-11-20

**Authors:** Maria Borragan, Clara D. Martin, Angela de Bruin, Jon Andoni Duñabeitia

**Affiliations:** ^1^Basque Center on Cognition, Brain and Language, Donostia, Spain; ^2^IKERBASQUE, Basque Foundation for Science, Bilbao, Spain; ^3^Facultad de Lenguas y Educación, Universidad Nebrija, Madrid, Spain

**Keywords:** inhbitory control, language production, bilingual experience, delayed auditory feedback, speech inhibition, lexical access

## Abstract

Multilinguals have to control their languages constantly to produce accurate verbal output. They have to inhibit possible lexical competitors not only from the target language, but also from non-target languages. Bilinguals’ training in inhibiting incongruent or irrelevant information has been used to endorse the so-called bilingual advantage in executive functions, assuming a transfer effect from language inhibition to domain-general inhibitory skills. Recent studies have suggested that language control may rely on language-specific inhibitory control mechanisms. In the present study, unbalanced highly proficient bilinguals completed a rapid naming multi-inhibitory task in two languages. The task assessed three types of inhibitory processes: inhibition of the non-target language, inhibition of lexical competitors, and inhibition of erroneous auditory feedback. The results showed an interaction between lexical competition and erroneous auditory feedback, but no interactions with the inhibition of the non-target language. The results suggested that different subcomponents of language inhibition are involved during bilingual language production.

## Introduction

Multilinguals have to manage different languages to control verbal speech on an everyday basis. They have to select the language that is needed at every specific moment and suppress interference from the situationally irrelevant languages. This mechanism is commonly referred to as language control and it has been associated with the use of a complex set of inhibitory control mechanisms (see Green, 1998; Kroll et al., 2008; Declerck and Philipp, 2015). Broadly speaking, inhibitory control refers to the suppression of interfering information or prepotent responses. In the influential framework published by Miyake et al. (2000), inhibition was proposed to be one of three separable components of executive functions (together with updating and shifting). However, a more recent framework (Miyake and Friedman, 2012) suggests that inhibition may not be a subcomponent but instead correlates perfectly with a “common executive function,” defined as the ability to maintain and use task goals and goal-related information.

Research has shown that language inhibition may be required during bilingual speech production (e.g., Rodriguez-Fornells et al., 2005; de Bruin et al., 2014) and comprehension (e.g., Macizo et al., 2010; Durlik et al., 2016), with the results from the former group of studies being more consistent than those from the latter. However, the mechanisms underlying language inhibition during bilingual speech production are not well understood yet, despite the importance of these inhibitory mechanisms in the debate on the so-called bilingual advantage in executive functions (see, among many others, Duñabeitia et al., 2014; Paap and Sawi, 2014; Bialystok, 2015; Duñabeitia and Carreiras, 2015; Sorge et al., 2017; see Lehtonen et al., 2018, for a recent review). In this respect, one important question is whether bilingual language inhibition is accomplished using the same mechanisms that are also used in non-linguistic inhibition tasks (i.e., a domain-general inhibitory mechanism; Jackson et al., 2001; Bialystok et al., 2008; Colzato et al., 2008; Linck et al., 2012; de Bruin et al., 2014) or whether mechanisms specific to linguistic inhibition are applied (Calabria et al., 2015; Branzi et al., 2016).

Furthermore, even within bilingual speech production, multiple types of linguistic conflict may be present that may be governed by different forms of inhibitory control mechanisms. The current study therefore assessed whether different types of interfering linguistic information are suppressed through a general inhibitory control mechanism or distinct mechanisms. To this end, we asked a group of highly proficient yet unbalanced Spanish-Basque bilinguals to complete a verbal production task either in their native language (Spanish) or in their non-native language (Basque), while parametrically manipulating other additional inhibitory demands (i.e., lexical inhibition and erroneous auditory feedback inhibition). While these three manipulations differ in many ways, they share one important component: the presence of interfering information that needs to be suppressed in order to correctly complete the task. In the current study, we explored the possible additive or interactive nature of the different types of interfering information in the context of a Rapid Automatized Naming (RAN) task (see Denckla and Rudel, 1974) in which we included several manipulations. The RAN task provides a unique opportunity to explore language-related interference at multiple levels, given that it taps into a fusion of linguistic, articulatory and attentional processes (see Cummine et al., 2014). In this line, it has been argued that over and above the obvious articulatory, motor and perceptual processes involved in the RAN, additional attentional, conceptual and phonological processes are required for successful performance (see Wolf and Bowers, 1999).

The first task manipulation concerned the use of the first or second language. It is widely assumed that multilingual speakers have to inhibit phonological and lexical competitors from the non-target language during speech production, so that speaking in one language requires *non-target language inhibition.* Green (1998) proposed an inhibitory control model in which multilinguals solve the conflict between languages through suppression of the representations from the non-target languages, while the representations from the target language are activated. Furthermore, the amount of inhibition needed to suppress the non-target language is argued to be related to language proficiency. In the case of a strong first language (L1) and weaker second language (L2), a relatively high level of inhibition of L1 is needed when speaking in L2. In contrast, when speaking in the stronger L1, less inhibition of the weaker L2 may be needed (although even in these circumstances, non-target language inhibition may be needed).

But over and above inhibiting the non-target language, both monolingual and bilingual speech production require a series of processes related with *lexical inhibition*. According to Levelt’s model of word production (Levelt et al., 1999), a series of automatic steps have to take place before a speaker voluntarily generates any word. First, she must identify a concept in the imagery system and activate the associated lexical representation(s). Then, she must select a suitable lexical item and inhibit the ones that share semantic, lexical, and syntactic properties with the target word. Finally, she must inhibit morphological and phonological competitors in order to retrieve the articulatory representation of the intended word. Hence, speakers have to inhibit possible lexical competitors in order to correctly produce the intended word (see Grainger and Jacobs, 1993; Abutalebi and Green, 2008; Philipp and Koch, 2009; Righi et al., 2010), and these lexical inhibitory mechanisms are qualitatively different from the non-target language inhibitory mechanisms insofar that the latter focus on the whole language system, while the former concentrate on the neighboring lexical representations.

But speech production does not exclusively rely on these two types of inhibitory mechanisms. During speech production, a speaker not only has to inhibit competitors at different levels of processing within the target and non-target languages, but she also has to trust her own auditory feedback to online monitor and control the articulatory output (Lee, 1950). Auditory feedback is a mechanism that helps to verify whether the current speech production is in agreement with the intention. In cases in which a mismatch in perceived, a correction mechanism operates at the level of production (see Burnett et al., 1998).

One interesting manipulation regarding speech monitoring is delayed auditory feedback (DAF), a technique that was initially developed to explore the importance of auditory feedback in speech production. The DAF is a technique in which speakers hear their own speech production through headphones, but with a short and artificially inserted lag between the actual production and its reproduction. Speech is normally inhibited using auditory feedback inhibition, which occurs online, with a very short delay. When the perception of speech is delayed – in this case artificially by playing back the sound with a delay – this auditory feedback inhibition becomes more costly and less efficient. Therefore, the auditory perceptual lag disturbs speech production, leading to disfluent utterances. The DAF technique facilitates understanding how production is achieved by exploring erroneous *auditory feedback inhibition* when such feedback is delayed and thus unreliable and even disturbing. In order to efficiently continue producing speech under DAF, speakers need to monitor the auditory feedback and inhibit the incorrectly timed input, while adjusting their utterances to the circumstances.

Some previous studies have suggested a relationship between auditory feedback and domain-general control processes. Adaptation to altered auditory feedback can be modulated by attentional load (e.g., Tumber et al., 2014; Scheerer et al., 2016) and networks mediating domain-general cognitive control may also be involved in feedback monitoring (Schiffer et al., 2015). Other studies, however, did not observe such a link between altered auditory feedback and domain-general inhibitory control (Martin et al., 2018) and have suggested that feedback control may rely on perceptual acuity to compensate for this perturbation during speech production (Villacorta et al., 2005; Martin et al., 2018). Yet, as auditory feedback is linguistic, a relationship may exist with language control, and we tested this idea in the current study.

The precise way in which language proficiency impacts speech production under DAF is still a matter of debate. It is assumed that until a certain proficiency level is acquired in a non-native language (L2), the impact of the DAF technique is larger in that language than in the native one (L1). Several studies have shown an interaction between language dominance and erroneous auditory feedback inhibition, reporting longer speech latencies in L2 than in L1 (e.g., Lee, 1950; Mackay, 1970; Van Borsel et al., 2005). These results are consistent with the idea that bilinguals need more inhibitory resources when using their weaker L2 because they have to suppress the dominant L1 (Green, 1998). However, once multilingual speakers acquire a higher level of proficiency in L2, erroneous auditory feedback inhibition seems to occur similarly for native and non-native languages, suggesting the control of incorrect auditory feedback is not exclusively related to nativeness in a language (e.g., Siegel et al., 1984; Kvavik et al., 1991; Fabbro and Darò, 1995). The participants in our study were highly proficient in both languages, but still unbalanced with a higher proficiency level in L1 than in L2. As such, our participants could follow the pattern of previous studies showing similar erroneous auditory feedback inhibition for L1 and L2 in bilinguals with a high proficiency level. Alternatively, the unbalanced proficiency levels may still lead to an interaction between auditory feedback and language.

In the current study, DAF was used to assess whether the demands of inhibiting the delayed feedback could cause a processing bottleneck for other inhibitory demands during speech production in the context of a RAN task. The RAN task was originally designed to assess reading competence by naming pictures as fast as possible (see Denckla and Cutting, 1999, for a review). This task requires not only lexical access, but also the inhibition of the competitors flanking the target image (i.e., the neighboring representations sharing some properties with the target item). In cases when the matrices are made of pictorial elements referring to the same semantic category (e.g., a picture of an animal flanked by other animals of different species), the inhibitory demands increase, making lexical access slower and costlier (see Oppenheim et al., 2010; Mahon and Caramazza, 2011; Runnqvist et al., 2012). Thus, the RAN task seems to be a perfect test scenario to explore how multiple levels of linguistic inhibitory demands could interact with each other during language production.

We created a multilingual RAN-like picture naming task where three types of language inhibition mechanisms could be required across conditions: non-target language inhibition, lexical inhibition of the preponderant responses and the competitors, and erroneous auditory feedback inhibition. We conceived an experimental design that allowed for observing how the system performs as a whole and whether these three variants of inhibitory processes at play during multilinguals’ speech production interact with each other. Firstly, highly proficient bilingual participants were asked to name the pictures of the RAN either in their native language (Spanish) or in their non-native language (Basque), thus requiring non-target language inhibition to complete the different trials of the RAN scenario.

Furthermore, an additional artificial inhibitory demand was included in the experimental design, aimed at mimicking some of the lexical inhibitory processes that need to be carried out by multilinguals while producing speech. Multilinguals and language learners have to inhibit preponderant words from the native language that may interfere with the correct utterance in the non-native language (e.g., a Spanish-English bilingual would have to inhibit the translation equivalent “casa” to produce the word “house”). This is an everyday, constant demand for bilinguals, and in the context of the current experiment, we artificially created a similar demand with the aim of recreating a natural aspect of bilinguals’ day-to-day interactions. We asked participants to substitute the name of certain pictures for that of some digits (e.g., say the word “two” when seeing the picture of a frog) in increasing order of difficulty, parametrically varying the number of to-be-replaced elements. Finally, auditory feedback demands were manipulated by including trials in which participants perceived their own speech without or with an artificial delay (DAF).

Thus, the aim of this study was to investigate how language inhibition works in multilingual speakers, particularly assessing whether distinct linguistic inhibitory processes that are at play during speech production interact with each other. By means of our multi-layered picture naming RAN-like task, we intended to tax the system and to evoke the use of large amounts of inhibitory resources, highlighting their independent effects and the interdependent interactions between them. The results of the current study will help us elucidating the extent to which language control mechanisms in multilingual speech production rely on a general mechanism of language control, or alternatively, on different subcomponents of language control. If the three types of inhibitory mechanisms interact with each other, this would suggest that language control relies at least partly on a shared inhibitory control mechanism. On the other hand, and in line with Sternberg’s Additive Factors Method Sternberg’s (1969, 1998, 2011), we argue that if the three types of interference manipulations show main effects that do no interact with each other, this would support the existence of independently operating processes. We would interpret a lack of interaction along the claims of the Additive Factors Method that has been successfully applied to visual object naming (see Sternberg, 1998), which endorses a view of additivity for functionally distinct processes that are separately modifiable (Sternberg, 2013). Thus, if we observe no interaction between non-target language inhibition (namely, the effect of naming items in L1 vs. in L2), erroneous auditory feedback inhibition (immediate feedback vs. delayed feedback), and lexical inhibition of competing representations, this would speak for a relative independence of the inhibitory components, in line with the idea of different inhibitory mechanisms underpinning language control (e.g., Calabria et al., 2012; Branzi et al., 2016). If it is the case that the systems work separately, our results should also shed new light on specific inhibitory processes applied within the language-related inhibitory system.

## Materials and Methods

### Participants

Fifty-sixunbalanced Spanish-Basque bilingual young adults from the University of the Basque Country took part in this experiment (highest degree obtained was high school for 12 participants, professional training for 8, university degree for 31, and postgraduate degree for 2 participants). Three participants were excluded from analyses due to a high error rate (more than 35% errors in each matrix). All participants (*M* age = 23 years, *SD* = 3 years; 33 females) were native Spanish speakers, who acquired Basque early in life (see Table 1) and were more exposed to Spanish than to Basque in their daily life (see Table 1). Their language proficiency was assessed using two tests (see de Bruin et al., 2017, for further details): a picture naming task in which they were asked to name 65 common objects in each of the two languages (see Table 1), and a personal interview with a native bilingual linguist who rated them on a 1-to-5 scale (5: native-like competence; 1: basic/no knowledge; see Table 1). In addition, participants were asked to rate their competence (in terms of reading, speaking, writing, and understanding) on a scale from 0 to 10 (see Table 1). All participants were right-handed and none were diagnosed with language disorders, learning disabilities, or auditory impairments. After the experiment, they were reimbursed for their time. This study was carried out in accordance with the recommendations of the international ethical guidelines approved by the BCBL Ethics Committee with written informed consent from all subjects. All subjects gave written informed consent in accordance with the Declaration of Helsinki. The protocol was approved by the BCBL Ethics Committee.

**Table 1:** Table showing the participants’ language profile.

	Spanish		Basque	
	*M*	*SD*	*M*	*SD*
Age of acquisition	0.5	1	3	1
Exposure	68.8	13	22.6	11
Picture naming test	64.6	0.8	52.1	6.6
Personal interview	5	0	3.9	0.3
Reading competence	9.6	0.7	7.4	1.2
Speaking competence	9.6	0.7	8.6	1.1
Writing competene	9.2	1.1	7.7	1.4
Understanding competence	9.6	0.7	8.6	1.3

*Mean and standard deviation are provided per language for age of acquisition (years), exposure (percentage of time exposed), picture naming test (number of correct items named in a scale from 0 to 65), personal interview (score in an ascending scale from 1 to 5), self-rated reading, speaking, writing, and understanding competence (scale 0–10).*

### Materials and Design

Pictures of concepts from two different semantic categories (animals and body parts) were used to create different matrices for the rapid naming task. The images were taken from the MultiPic database (Duñabeitia et al., 2018). While non-target language activation has often been studied with words that are similar between two languages (e.g., cognates), studies using non-cognates have also observed activation and inhibition of the non-target language (e.g., de Bruin et al., 2014). To avoid effects of cognate status, we explicitly avoided the use of concepts associated with cognate words, and all the items selected for the two categories had names that were non-cognates between Spanish and Basque, lacking substantial orthographic or phonological overlap between languages. The picture names were matched on syllable length, number of phonemes, and frequency of use between languages (see Table 2).

**Table 2:** Means (M) and standard deviations (SD) of the number of syllables, phonemes and of the frequency of use (in number of appearances per million) of the common names used in the task, as obtained from the E-Hitz (Perea et al., 2006) and B-Pal (Davis and Perea, 2005) databases.

	Animals				Body parts			
	Spanish		Basque		Spanish		Basque	
	*M*	*SD*	*M*	*SD*	*M*	*SD*	*M*	*SD*
Syllables	2.5	0.5	2	0	2	0.6	2	0.6
Phonemes	5	0.9	4.3	0.5	4	0.8	4	1.4
Word frequency	20.6	21.5	33.6	24.1	136.8	132.3	116.3	106.1

**FIGURE 1:**
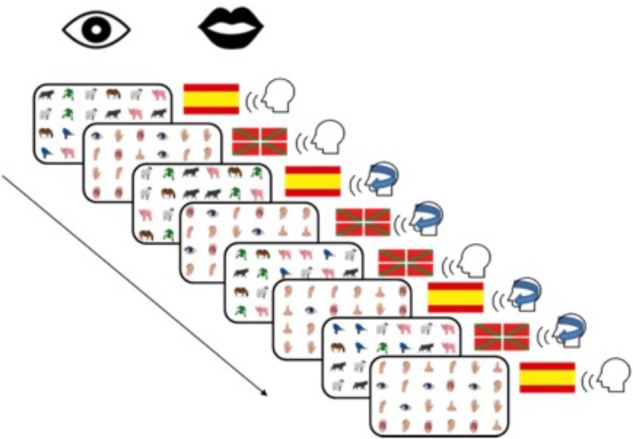
Overview of the eight different blocks completed by each participant. Language block order (Basque/Spanish) was counterbalanced across participants, and the order of presentation of the rest of conditions within each language block was randomized.

The structure of the experiment and the order of conditions were as follows. Each participant completed eight blocks. Four blocks were completed in Spanish and four in Basque. Furthermore, two of the four blocks were completed without DAF and the other two with DAF (i.e., two of the four blocks) for each language. Each semantic category occurred once in each of these conditions, so that each participant completed the same semantic category twice in each language, once with and once without DAF (see Figure 1). The blocks were distributed over the experiment so that in the first half of the experiment, some participants completed all body part blocks in Spanish (both with DAF and without DAF) and all animal blocks in Basque. In the second half of the experiment, these participants would then complete all animal blocks in Spanish and all body part blocks in Basque. For other participants, this order was reversed so that the language in which each semantic category was named first was counterbalanced. Within each half of the experiment, the order of languages and DAF condition was randomized so that some participants started in Spanish and some in Basque.

Furthermore, each block was composed of 4 matrices that were always completed in the language of that block. Each matrix included 24 pictures (i.e., each of the 6 individual items was repeated four times; see Appendix 1) aligned in columns and rows and arranged at random. Participants were asked to name the pictures of the first matrices of each block normally. Then, in the subsequent 3 matrices, participants were asked to replace the name of certain items with the name of some numbers. In the second matrix of each block, the name of one item (i.e., one animal or one body part) had to be consistently replaced throughout the completion of the matrix with the name of the first cardinal number (e.g., replace the word “frog” with the word “one”). In the third matrix, the names of two pictures had to be substituted by the names of two digits. Finally, in the fourth matrix of each block, participants were asked to replace the names of three different items with those of three digits. The specific rules for the replacements in each matrix were presented to the participants at the beginning of each trial. The blocks were counterbalanced for language and the presence or absence of DAF, but lexical substitutions were always presented in the same order following an increasing order of difficulty from 0 to 3.

### Procedure

Participants were tested individually in a soundproof room. They were seated at a distance of about 45 cm from a laptop with a 13-inch screen, where all the stimuli were presented using Experiment Builder (SR-Research, Ontario, Canada). The same software was also used to collect the verbal responses, which were recorded from the onset of the presentation of each matrix to the moment in which the participant pressed the space bar to indicate that she had finished naming the items. Participants wore a headset throughout the experiment and they were instructed to name the items of each matrix as if they were reading a text (left to right, top to bottom), as fast as they could and trying not to make errors. Delayed auditory feedback time was set to 200 ms for the DAF condition in accordance with previous studies (Stuart et al., 2002; Hashimoto and Sakai, 2003) and to 0 ms for the immediate (no-DAF) condition. To this end, a SmallTalk device (Casa Futura Technologies, Colorado, United States) set at 80 dB was used.

Each matrix in each block was preceded by a screen specifying the instructions and conditions about the auditory feedback (delayed vs. immediate), the assigned language (L1 vs. L2), and the number of lexical substitutions that they had to do (0, 1, 2, or 3). Participants were instructed whether there would be a delay in auditory feedback to avoid the disruptive effect of the delay being larger at the beginning of the task due it being a surprise. Participants were familiarized with the pictures’ names before performing the rapid naming task in the two languages and they practiced the lexical substitution with fruit matrices before the experiment started.

### Data Analysis

Data were analyzed in two ways. Firstly, we performed a three way ANOVA testing the effects of Language (L1| L2), Auditory Feedback (immediate| delayed), and Lexical Replacements (namely, the number of substitutions: 0| 1| 2| 3) on the naming latencies. As we aimed to examine whether different types of linguistic conflict interacted or not, we furthermore analyzed the data using Bayesian analysis. In the case of a null effect, a *p-*value can only say that there was no evidence for an effect, but it does not support the absence of an effect. By reporting Bayes Factors (BF), we show the ratio of the probability that the data were observed under the null hypothesis (e.g., “no interaction between auditory feedback and language”) vs. the probability of observing the data under the alternative hypothesis (e.g., “an interaction between auditory feedback and language”). For instance, a BF_01_ of 5 indicates that the observed data were five times more likely to have occurred under the null than alternative hypothesis. Bayesian analyses were conducted with JASP 0.8.5 using Bayesian repeated measures ANOVA with 100,000 samples. As we were interested in the interactions between the three different manipulations, we compared the model with the three main effects of Language, Auditory Feedback, and Lexical Replacements to a model including those three main effects plus, (a) the interaction between Language and Auditory Feedback; (b) the interaction between Language and Lexical replacements; and (c) the interaction between Auditory Feedback and Lexical Replacements.

## Results

We exclusively focused on the naming latencies given that the average number of errors was fairly low (*M* = 1.05 errors per matrix, *SD* = 1.30; range: 0.17–2.17). Besides, it is likely that any effects of production errors are also observable in the naming latencies since participants corrected themselves when making a mistake, thus requiring more time to complete the matrix.

**FIGURE 2:**
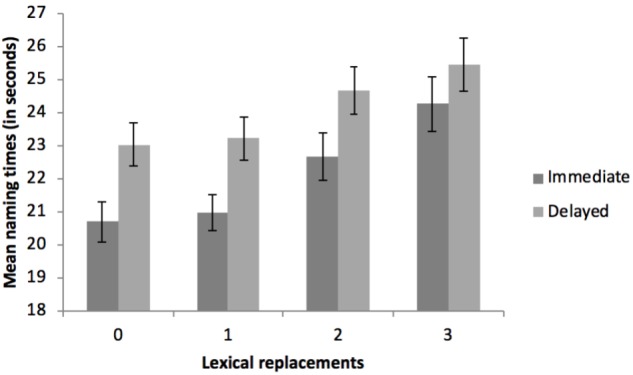
Mean naming times (in seconds) as a function of the number of lexical replacements (0, 1, 2 or 3) and the auditory feedback conditions (immediate vs. delayed). Error bars refer to the standard error (SE) of each mean.

**FIGURE 3:**
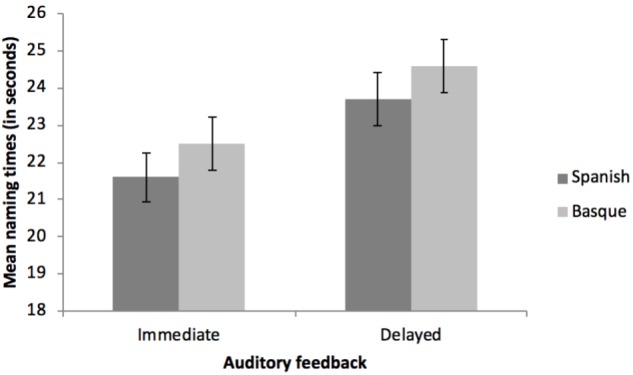
Mean naming times (in seconds) as a function of the language (Spanish and Basque) and the auditory feedback conditions (immediate and delayed). Errors bars refer to the standard error (SE) of each mean.

**FIGURE 4:**
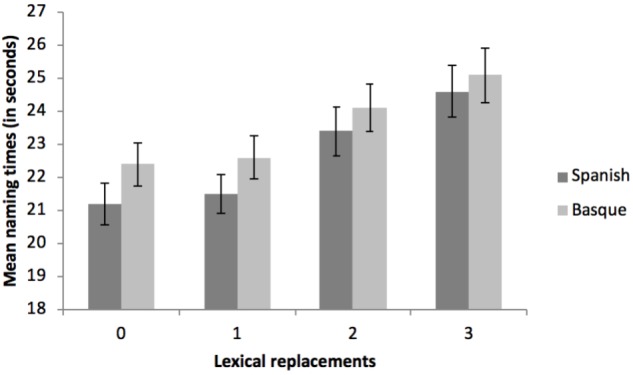
Mean naming times (in seconds) as a function of the language (Spanish and Basque) and the lexical replacements (0, 1, 2, or 3). Errors bars refer to the standard error (SE) of each mean.

The three main effects were significant. For the effect of Language, blocks that were named in Basque (L2, non-native language) yielded longer reaction times (*M* = 23.61 s, *SD* = 5.20) than blocks completed in Spanish (L1, native language; *M* = 22.67 s, *SD* = 4.93), *F*(1, 52) = 9.30, *p* < 0.004, $\eta_{p}^{2}$ = 0.152^1^. Regarding the effect of Auditory Feedback, blocks that had to be named under the DAF condition required more time (*M* = 24.18 s, *SD* = 5.19) than blocks that did not include any DAF (*M* = 22.10 s, *SD* = 4.95, *F*(1, 52) = 57.26, *p* < 0.001, $\eta_{p}^{2}$ = 0.524. Finally, the main effect of Lexical Replacements showed an increase in the naming latencies as a function of the number of words that had to be substituted, *F*(3, 156) = 38.73, *p* < 0.001, $\eta_{p}^{2}$ = 0.427, ranging from trials requiring no replacements (*M* = 21.88 s, *SD* = 4.60 to trials requiring 3 substitutions (*M* = 24.87 s, *SD* = 5.91) (see Table 3 and Figure 2 for details). There was a significant interaction between Auditory Feedback and Lexical Replacements, *F*(3, 156) = 3.67, *p* = 0.014, $\eta_{p}^{2}$ = 0.066, such that the effect of the DAF diminished as the number of replacements increased (see Figure 2). Nonetheless, and in spite of the decreasing magnitudes of the effect of the auditory feedback with the increased lexical replacement demands, this effect was always significant [No substitutions: *t*(52) = 5.28, *p* < 0.001, Cohen’s *d =* 0.726*;* 1 substitution: *t*(52) = 6.95, *p* < 0.001, Cohen’s *d =* 0.956*;* 2 substitutions: *t*(52) = 6.76, *p* < 0.001, Cohen’s *d* = 0.929*;* 3 substitutions: *t*(52) = 2.83, *p* < 0.006, Cohen’s *d* = 0.390). Importantly, there was no interaction between Language and Auditory Feedback, *F*(1, 52) = 0.20, *p* = 0.653, $\eta_{p}^{2}$ = 0.004 (see Figure 3), or between Language and Lexical Replacements, *F*(3, 156) = 1.32, *p* = 0.270, $\eta_{p}^{2}$ = 0.025 (see Figure 4), nor was there a three-way interaction between all the factors, *F*(3, 156) = 0.41, *p* = 0.750, $\eta_{p}^{2}$ = 0.008.

**Table 3:** Descriptive statistics of the mean naming latencies (in seconds) across participants in each condition.

	Lexical replacements							
	0		1		2		3	
	Mean	*SD*	Mean	*SD*	Mean	*SD*	Mean	*SD*
**NATIVE LANGUAGE (SPANISH)**								
Immediate	20.10	3.91	20.11	3.92	22.37	5.28	24.11	5.65
Feedback								
Delayed	22.41	5.02	22.60	4.58	24.50	5.28	25.18	5.86
Feedback								
**NON-NATIVE LANGUAGE (BASQUE)**								
Immediate	21.33	4.94	21.32	4.24	22.99	5.33	24.47	6.35
feedback								
Delayed	23.68	4.53	23.85	5.21	24.83	5.19	25.72	5.81
Feedback								

We then conducted Bayesian analyses in which we compared models including the main effects only to the model including the main effects plus the interaction of interest. For the model including the interaction between Language and Lexical Replacements, the BF_01_ was 37.85 (± 2.65%), suggesting that model without an interaction fits the data around 38 times better than a model with this interaction term included. Similarly, for the model including the Language × Auditory Feedback interaction, the BF_01_ was 8.76 (± 1.71%), suggesting that the model without such interaction accounts for the data nearly 9 times better than the model with the interaction. Thus, both analyses suggested that there was no interaction between Language and Auditory Feedback or Lexical Replacements. Regarding the interaction between Lexical Replacements and Auditory Feedback, while the *p-*value showed a significant interaction, the Bayes Factor analysis showed some evidence against an interaction with a BF_01_ of 4.25 (± 4.18%).

## Discussion

The aim of the study was to explore how distinct types of language inhibition that are typically needed by multilingual speakers to efficiently produce speech (namely, non-target language inhibition, inhibition of lexical competitors, and inhibition of erroneous auditory feedback) interact with each other during completion of a rapid naming task. Our main interest was to examine whether these different processes rely on the same linguistic inhibitory system or whether, alternatively, several types of different and independent linguistic inhibitory mechanisms underlie each of the distinct processes. To this end, we designed a highly demanding rapid naming task to allow us to observe how the inhibitory system(s) work(s) while multitasking.

In accordance with previous findings and as predicted, our results revealed main effects in all the three variables of interest. Firstly, participants exhibited longer naming times overall in L2 (Basque) as compared to L1 (Spanish), in line with the bulk of preceding evidence at this regard (e.g., Meuter and Allport, 1999; Costa and Santesteban, 2004). Second, longer naming latencies were also observed under DAF conditions as compared to immediate feedback conditions (see Lee, 1950; Mackay, 1970; Van Borsel et al., 2005). Finally, regarding the effect of replacing a preponderant response with a different lexical label according to newly learned rules, we found that naming times increased as a function of the number of replacements that were required. These effects suggest that the current test scenario readily tapped into a set of inhibitory mechanisms whose role and degree of implication were more prominent as the task demands increased (see Wickens, 2002).

Interestingly, only two of the constructs associated with different inhibitory demands interacted with each other in the classic factorial analysis of variance (although the Bayes Factor actually provided some evidence *against* this interaction). The negative impact of the DAF partially decreased as the lexical competition increased (namely, as the need for controlling and inhibiting a preponderant response increased), potentially suggesting that both may tap into similar inhibitory resources. This interaction could also be understood in terms of a plateau effect in the three-replacement condition when the maximal taxation of cognitive resources is reached. However, none of these effects significantly interacted with the language at use (native vs. non-native), and the relative independence of this effect speaks for a certain degree of separation or autonomy of the different types of inhibitory mechanisms that multilinguals may use and require.

Crucially, the language (L1 or L2) did not interact with either lexical competition or auditory feedback alteration. This suggests that inhibitory mechanisms used to suppress the non-target language are not identical to the inhibition mechanisms used to accomplish suppression of erroneous auditory feedback or lexical competitors. The latter manipulation may also have introduced an increase in working memory load, which could explain the main effect of lexical competitors that was observed. However, beyond the working memory component related to remembering which replacement to use, the task also required inhibition of the word that could not be used (or resistance to the interference created by this salient representation). Thus, if the inhibitory component of lexical competition is related to the type of control used in language inhibition, we should have observed an interaction. Instead, and in line with Sternberg’s Additive Factors Method Sternberg’s (1998; 2013), the additive nature of these effects and the demonstration that they are independently modifiable, support the idea of a functional difference between them.

Previous studies have already suggested that language inhibition may be based on its own specific and independent resources (see Abutalebi and Green, 2008; Calabria et al., 2012; Blanco-Elorrieta and Pylkkänen, 2016; Branzi et al., 2016). Our results are in line with this view, suggesting that the inhibition of the non-target language is likely to be managed through a set of inhibitory resources that are not shared or required by other tasks. These results follow what Shell et al. (2015) showed in a similar study manipulating inhibitory control demands in a picture-word interference task that also involved a language-switching paradigm. No interaction between the effects of the lexical competitors and the effects of the language at use was found in this study either, suggesting that the underlying processes may not require overlapping or shared inhibitory mechanisms, ultimately suggesting that multilingual language control could use a highly specific and independent inhibitory mechanism for non-target language inhibition.

On the other hand, our data are less compatible with previous studies suggesting that language inhibition is at least partly related to the inhibition mechanisms used in other (non-verbal) tasks (e.g., Linck et al., 2012; de Bruin et al., 2014). However, these studies have typically used a language switching paradigm, which may place additional demands on language inhibition and/or may require a different form of inhibition. For instance, while our task required more global, proactive inhibition of the non-target language, language switching may make additional use of local, reactive inhibition mechanisms (Green, 1998). Considering the design of the current experiment, it was not possible to examine more short-lived effects of inhibition at the level of individual items. Future experiments will need to examine whether different tasks eliciting stronger and/or more local effects of language inhibition show connections between different types of language inhibition.

The absence of an interaction between inhibition of the delayed, erroneous auditory feedback and inhibition of the non-target language is line with a previous study (Martin et al., 2018) showing that the type of inhibition applied to compensate for the presence of altered feedback does not correlate with other types of inhibitory processes. It is furthermore worth noting that while some preceding studies have shown an interaction between the effects of DAF and those of language dominance (see Lee, 1950; Mackay, 1970; Van Borsel et al., 2005), such an interaction has not been found in samples of highly proficient bilinguals. For instance, Fabbro and Darò (1995) conducted a study with highly proficient interpreters performing under DAF conditions and found no interaction between the critical variables of interest. The participants in our study were highly proficient in both languages. In line with preceding evidence showing that highly proficient bilinguals rely on different language-selection mechanisms than low proficient bilinguals (e.g., Costa and Santesteban, 2004; Costa et al., 2006), increased proficiency in the non-native language could make the reliance on erroneous auditory feedback more similar to that of the native language. If so, the set of inhibitory mechanisms that are used to partial out the negative effect of incorrect (altered or delayed) auditory feedback could be similar in L1 and L2. This is precisely what we found in the current study, suggesting that in highly proficient bilinguals, the underlying processes responsible to monitor erroneous auditory feedback and inhibit the potentially disturbing incorrect feedback may not be linguistic in essence, and that it may instead correspond to a specific type of mechanisms that are linked to perceptual acuity.

This study suggests that multilinguals do not rely on one unique inhibitory system to produce speech in one of the known languages, but rather that they rely on a set of different mechanisms that operate separately. As said, the interpretation that additive factors can reflect independent processes is in line with Sternberg’s Additive Factors Method Sternberg (1998). This approach has been used frequently to study the independence of and similarities between different processes involved in inhibitory control (e.g., Los, 2004; van den Wildenberg and van der Molen, 2004). However, other studies have questioned the reliability of inferring underlying processes from patterns seen in response time data. Stafford and Gurney (2011) showed that both models with discrete stages and continuous models with simultaneously run, interacting processes could mimic additive factors. Any inference with respect to distinct vs. interacting processes based on additive factors should thus be interpreted with caution. In line with Sternberg (2013) response to Stafford and Gurney, we therefore interpret our data as *supporting* rather than unequivocally implying distinct inhibitory control processes.

The three manipulations used in our study were not only tapping into inhibitory control, but are also likely to recruit other forms of cognitive control (e.g., working memory or rule learning, in the case of the manipulation involving lexical replacements). While it is perfectly possible that the three manipulations were different enough not to recruit a shared inhibitory control mechanism, it should be considered that the three of them required the inhibition of interfering information (in the form of another language, lexical items, or erroneous auditory feedback). We believe that if different types of inhibitory control are governed by a common inhibitory control mechanism, these three specific forms of resistance to linguistic interference would be expected to tap into this general mechanism.

Our results open up the possibility to think about how the inhibitory system is further divided into domain-specific sub-mechanisms that do not necessarily work at par. These distinct mechanisms should be explored in more detail in order to better understand how they work and relate to each other in an interactive fashion, and specifically, how they are used by multilinguals to efficiently face the highly demanding communicative scenarios they encounter in their daily life. Furthermore, brain imaging techniques could be used to shed more light on the possible differences between or overlap in the spatial and temporal characteristics of different types of inhibitory control. We tentatively propose that the debate on the generality or specificity of the language-related inhibitory mechanisms should be moved to a new arena, leaving aside the simplistic dichotomy between language-specific inhibition and domain-general inhibition. The current study suggests that the different inhibitory processes that mediate multilingual speech production are somewhat independent from each other, probably referring to diverse inhibitory modules. Some of these mechanisms, such as the inhibitory control of erroneous auditory feedback and the inhibition of competing lexical representations, could tap into similar inhibitory resources. However, other mechanisms, such as the set of inhibitory processes applied to control for the interference from the non-target language, seem to be independent. Together, these results make us think of a series of inhibitory modules that go beyond a unitary conception of language-specific inhibitory mechanisms.

In sum, these findings demonstrate that some of the mechanisms related to language control require allocating particular and independent inhibitory resources that remain unaffected by the concurrent requirement of other inhibitory mechanisms. This suggests that multilingual language control builds on a set of specific inhibitory mechanisms that are not shared by other cognitive or even other language processes. Future studies will help us elucidating the precise nature and role of these seemingly independent inhibitory processes, and the way in which they are acquired, developed and trained in multilingual contexts.

## Author Contributions

MB, CM, and JD designed the study. MB and JD created the materials and experimental protocols. MB collected the data, analyzed the data under the supervision of all the authors, and drafted the manuscript. All authors provided critical comments on the manuscript before submission.

## Conflict of Interest Statement

The authors declare that the research was conducted in the absence of any commercial or financial relationships that could be construed as a potential conflict of interest.

## Footnotes

1 To further examine the role of non-target language inhibition in L1 vs. L2, we examined effects of language order. If L1 is inhibited strongly during L2 production, L1 responses should be relatively slow after an L2 task compared to L2 after L1. Participants always named one semantic category in one language in the first half of the experiment and in the other language in the other half. There was a significant effect of task half [*F*(1, 52) = 8.77, *p* = 0.005, $\eta_{p}^{2}$ = 0.144], reflecting that responses were faster in the first half (*M* = 22.70 s, *SD* = 4.30) than in the second half (*M* = 23.58 s, *SD* = 4.50) of the experiment. However, while the difference between task half was numerically larger for Spanish (*M* = 1.23; *SD* = 2.62) than Basque (*M* = 0.53; *SD* = 3.95), task half did not interact significantly with language [*F*(1, 52) = 1.00, *p* = 0.321, $\eta_{p}^{2}$ = 0.019].

## Appendix 1

Examples of matrices from the two different categories (animals and body parts) used in the experiment, together with the expected names to be said in Spanish and Basque (the English translations are as follows: toro, bull; rana, frog; oveja, sheep; caballo, horse; cerdo, pig; pájaro, bird; ojo, eye; mano, hand; boca, mouth; pie, foot; nariz, nose; oreja, ear) under the no replacement condition.
